# Sensitivity of US and FNAC for staging of the axilla in patients presenting with symptomatic breast cancer

**DOI:** 10.1186/bcr3763

**Published:** 2015-11-05

**Authors:** Diane Lister, Miaad Al-Attar, Elizabeth Denton, Lisa Grosvenor, Gayle McDonald, Dave Purnell

**Affiliations:** 1 Breast Care Centre, UHL NHS Trust, Leicester, UK

## Introduction

We investigated our sensitivity for axillary node staging, in patients presenting with symptomatic breast cancer from January to December 2012.

## Methods

Of 430 patients identified, 288 had first-line surgical treatment, 63 had neoadjuvant therapy first. Seventy-nine women were unfit for surgery, had less aggressive evaluation of the axilla and were excluded from sensitivity calculations. US axilla ± FNA were performed at presentation. Nodal disease prevalence, sensitivity for diagnosis and the NPV of our tests were calculated. In the neoadjuvant cases, pretreatment nodal status was not accurately known.

## Results

The prevalence of nodal metastases in our surgery first cases was 43% (123/288). Twenty-four per cent of cases were micrometastases (29/123). US sensitivity for macrometastases was 51% (48/94); 41% including micrometastases (50/123). FNA sensitivity for macrometastases was 38% (36/94; 35 results C5); 30% (37/123) including micrometastases. Combining all groups, FNA was definitive (C5 or C2) in 90% (134/149) of cases. The NPV of imaging was 65% (137/210); 75% (137/183) with micrometastases excluded. The NPV of a C1/2 result was 72% (28/39) giving a false negative FNA rate of 28%. Of neoadjuvant cases, FNA was positive in 60% (38/63; 35 results C5), giving a minimum disease prevalence and diagnostic sensitivity of 60%. Combining both groups, nodal disease prevalence lies between 46% (161/351) and 53% (186/351). FNA sensitivity is between 48 and 57% for macrometastases (75/157; 75/132); and 40−46% including micrometastases (75/186; 75/161).

## Conclusion

Axillary staging depends on both US sensitivity and FNAC technique. US sensitivity is adversely affected by micrometastases. In our symptomatic patients, FNA sensitivity for macrometastases lies between 48 and 57%.

